# Palmitate and insulin counteract glucose-induced thioredoxin interacting protein (TXNIP) expression in insulin secreting cells via distinct mechanisms

**DOI:** 10.1371/journal.pone.0198016

**Published:** 2018-05-29

**Authors:** Madhura Panse, Oliver Kluth, Estela Lorza-Gil, Gabriele Kaiser, Eckhard Mühlbauer, Annette Schürmann, Hans-Ulrich Häring, Susanne Ullrich, Felicia Gerst

**Affiliations:** 1 University of Tübingen, Department of Internal Medicine, Division of Endocrinology, Diabetology, Vascular Medicine, Nephrology and Clinical Chemistry, Tübingen, Germany; 2 German Center for Diabetes Research (DZD), München-Neuherberg, Germany; 3 German Institute of Human Nutrition Potsdam-Rehbruecke, Department of Experimental Diabetology, Potsdam-Rehbruecke, Germany; 4 Institute for Diabetes Research and Metabolic Diseases of the Helmholtz Center Munich at the University of Tübingen (IDM), Tübingen, Germany; Broad Institute, UNITED STATES

## Abstract

Glucose and palmitate synergistically stimulate insulin secretion, but chronically elevated they induce apoptotic β-cell death. The glucotoxic effect has been attributed, at least partly, to the upregulation of the oxidative stress marker thioredoxin interacting protein (TXNIP). Palmitate downregulates TXNIP expression, the functional significance of which is still under debate. This study examines the mechanism and consequence of palmitate-mediated TXNIP regulation in insulin secreting cells. Palmitate (600 μM) reduced TXNIP mRNA levels in isolated human and mouse islets independently of FFAR1/GPR40. Similar effects of palmitate were observed in INS-1E cells and mimicked by other long chain fatty acids. The lowering of TXNIP mRNA was significant already 1 h after addition of palmitate, persisted for 24 h and was directly translated to changes in TXNIP protein. The pharmacological inhibition of palmitate-induced phosphorylation of AMPK, ERK1/2, JNK and PKCα/β by BML-275, PD98059, SP600125 and Gö6976, respectively, did not abolish palmitate-mediated TXNIP downregulation. The effect of palmitate was superimposed by a time-dependent (8 h and 24 h) decline of TXNIP mRNA and protein. This decline correlated with accumulation of secreted insulin into the medium. Accordingly, exogenously added insulin reduced TXNIP mRNA and protein levels, an effect counteracted by the insulin/IGF-1 receptor antagonist linsitinib. The inhibition of PI3K and Akt/PKB increased TXNIP mRNA levels. The histone deacetylase (HDAC1/2/3) inhibitor MS-275 completely abrogated the time-dependent, insulin-mediated reduction of TXNIP, leaving the effect of palmitate unaltered. Acute stimulation of insulin secretion and chronic accentuation of cell death by palmitate occurred independently of TXNIP regulation. On the contrary, palmitate antagonized glucose-augmented ROS production. In conclusion, glucose-induced TXNIP expression is efficiently antagonized by two independent mechanisms, namely via an autocrine activation of insulin/IGF-1 receptors involving HDAC and by palmitate attenuating oxidative stress of β-cells.

## Introduction

Obesity-dependent type-2 diabetes is associated not only with chronically elevated blood glucose but also with an elevation of serum non-esterified fatty acids (NEFAs) concentration [1]. Although both glucose and NEFAs acutely stimulate insulin secretion, chronically elevated, they exert a negative effect on the β-cell function [2]. Glucotoxicity has been linked to increased oxidative stress and mitochondrial dysfunction resulting in apoptosis [3–5]. Apoptotic β-cell death induced by long chain saturated fatty acids such as palmitate is linked to the stimulation of stress kinases and ER stress [6–9]. Multiple cellular defense mechanisms in form of superoxide dismutase, catalase, glutathione peroxidase, peroxiredoxin, glutathione S-transferase and thioredoxin can detoxify the endogenous oxidants and reduce oxidative stress [10]. The antioxidative capacity of thioredoxin is antagonized by the cellular protein thioredoxin interacting protein (TXNIP) [11].

The regulation of TXNIP expression by glucose has been extensively studied. TXNIP gene transcription is activated by the transcription factor carbohydrate-response element-binding protein (ChREBP) which binds to the TXNIP promotor [12, 13]. The nuclear-cytoplasmic shuttling of ChREBP as well as its binding to DNA are regulated by PKA- and AMPK-mediated phosphorylations. Phosphorylated ChREBP is localized in the cytosol, while a loss of phosphorylation triggers its nuclear accumulation and DNA binding [14, 15]. Additionally, in pancreatic β-cells the nuclear translocation of ChREBP is also initiated upon elevation of cytosolic calcium concentration driven by glucose metabolism [16]. Thus, under high glucose, there is an increased nuclear accumulation and DNA binding ability of ChREBP due to the low AMPK activity. The nuclear ChREBP forms a heterodimer with MondoA and binds to the TXNIP promoter. Subsequently, the histone acetyltransferase p300 is recruited which leads to initiation of TXNIP transcription [13].

Long chain fatty acids counteract glucose-induced TXNIP expression in pancreatic β-cells [17]. However, high concentrations of fatty acids induce apoptotic cell death and accentuate rather than reduce glucotoxicity, in spite of TXNIP downregulation. The present study was undertaken to understand the implications of apparent contradictory situations, namely: palmitate-mediated downregulation of the oxidative stress inducer TXNIP, under conditions of lipotoxicity. To this end, the signalling pathways activated by palmitate in counteracting glucotoxicity-mediated upregulation of TXNIP, were examined in detail. The results will help decipher the physiological role of TXNIP regulation under glucolipotoxic conditions.

## Materials and methods

### Cell culture

INS-1E cells, kindly provided by C.B. Wollheim and P. Maechler (University of Geneva, Geneva, Switzerland), were cultured in RPMI1640 medium (Lonza, Basel, Switzerland) containing 11 mM glucose and supplemented with 10% FCS (Serva, Heidelberg, Germany), 10 mM HEPES, 2 mM L-glutamine, 1 mM Na-pyruvate and 10 μM 2-mercaptoethanol without addition of antibiotics.

### Isolation of mouse islets and culture of human and mouse islets

Isolated human islets were obtained from European Center for Islet Transplantation (ECIT, Geneva, Switzerland and Milan, Italy) and were cultured in CMRL1066 containing 5 mM glucose, 2 mM L-glutamine, 10 mM HEPES and supplemented with 10% FCS. Islets from 6–10 months old C57BL/6 mice (WT and *Ffar1*^(-/-)^) were isolated using collagenase digestion (1 mg/ml, #NB8, Serva, Heidelberg, Germany) and cultured overnight in INS-1E cell medium without 2-mercaptoethanol.

The experiments with human islets were approved by local Ethic Commission (Ethik-Kommission an der Medizinischen Fakultät er Eberhards-Karls-Universität und am Universitätsklinikums Tübingen, Gartenstrasse 47, 72074 Tübingen, Vorsitzender Prof. Dr. med. Luft, No. 089/2017BO1, 18.01.2017). Isolation of mouse islets was conducted in accordance with the accepted standard of animal care and was approved by the local authorities (Mitteilung (Notification) after §4 Abs. 3 TierSchG from 21.02.2014 and 19.10.2016 to the Regierungspräsidium Tübingen, Referat 35, Konrad Adenauer Strasse 20, 72072 Tübingen through the Animal Welfare Officer of the University of Tübingen).

### Treatment with test substances

A stock solution (100 mM) of palmitic acid (#P0500, Sigma-Aldrich, Munich, Germany) was prepared in DMSO and was added at a final concentration of 6 mM to FCS (containing 5% serum albumin) or modified Krebs-Ringer buffer (KRB) supplemented with 5% BSA. The GPR40 agonist TUG-469 (a kind gift from Prof. T. Ulven, Southern University of Denmark, Denmark) was applied at concentrations of 3–10 μM in culture medium supplemented with 1% FCS (containing 0.05% BSA) [2, 18]. The AMPK activator AICAR (#BML-EI-330, Enzo Life Sciences, Farmingdale, NY, USA), the PI3K inhibitor LY294002 (#440202, Merck Millipore, Burlington, MA, USA), the JNK inhibitor SP600125 (#S5567, Sigma-Aldrich, Munich, Germany), the ERK1/2 inhibitor PD98059 (#51300, Merck Millipore, Burlington, MA, USA), the AMPK inhibitor BML-275 (#BML-EI-369, Enzo Life Sciences, Farmingdale, NY, USA), the PKCα/β inhibitor Gö6976 (#365250, Merck Millipore, Burlington, MA, USA), the HDAC1/2/3 inhibitor MS-275 (#EPS002, Sigma Aldrich, Munich, Germany) and Actinomycin D (#A1410, Sigma-Aldrich, Munich, Germany) were dissolved in DMSO. Inhibitors were applied 1 h prior to palmitate and kept throughout the treatment.

### Insulin measurements by radioimmunoassay (RIA)

Insulin secretion was performed in static incubations with KRB containing (in mM): 135 NaCl, 4.8 KCl, 1.2 MgSO_4_, 1.3 CaCl_2_, 1.2 KH_2_PO_4_, 5 NaHCO_3_, 10 HEPES and 5 g/l BSA (#A8806, fatty acid free; Sigma-Aldrich), pH 7.4. The cells were preincubated for 1 h at 2.8 mM glucose and incubated for another hour in the presence of test substances. Secreted insulin and insulin content of the cells after extraction in acid ethanol (1.5% (v/v) HCl in 75% (v/v) ethanol) were measured using radioimmunoassay (#RI-13K, Millipore, Billerica, MA, USA).

### RNA isolation and qRT-PCR

INS-1E cells, mouse and human islets were lysed, total RNA isolated and contaminating DNA degraded by DNase digestion (#74106, RNEasy kit, Qiagen, Hilden, Germany or #740955, Nucleospin RNA isolation kit, Macherey Nagel, Düren, Germany). RNA integrity was evaluated using a bioanalyser (Agilent Technologies, Santa Clara, CA, USA) and 0.1–0.5 μg of RNA (RIN>8.5) was transcribed into cDNA using a recombinant reverse transcriptase (#4897030001, Transcriptor first strand cDNA synthesis, Roche Diagnostics, Rotkreuz, Switzerland). Quantitative PCR was performed with the LightCycler480 system (Roche Diagnostics). The sequences of specific primers (Invitrogen, Carlsbad, CA, USA) are listed in S1 Table. The values of target gene expressions relative to the house keeping gene (*Rps13*) are plotted as ΔCt, the effect of treatment as ΔΔCt.

### Western blotting

INS-1E cells were lysed in a buffer containing 125 mM NaCl, 1% (v/v) Triton X-100, 0.5% Na-deoxycholate, 0.1% SDS, 10 mM EDTA, 25 mM HEPES, pH 7.3, 10 mM NaPP, 10 mM NaF, 1 mM Na-vanadate, 10 μg/ml pepstatin A, 10 μg/ml aprotinin and 0.1 mM PMSF as described previously [19]. Proteins (30–50 μg) were subjected to SDS-PAGE and blotted on nitrocellulose membranes (Hahnemühle, Dassel, Germany). Membranes were incubated overnight with primary antibodies against TXNIP (#K0205-3, MBL International, Woburn, MA, USA), P-Thr172-AMPKα (#2535, Cell Signaling Technology, Danvers, MA, USA), AMPKα1 (#27947, Upstate Biotechnology from Merck Millipore, Darmstadt, Germany), P-Thr183/Tyr185-JNK (#9251, Cell Signaling Technology), P-Ser73-cJUN (#9164, Cell Signaling Technology), P-Ser473-AKT (#9271, Cell Signaling Technology), AKT (#9272, Cell Signaling Technology), cleaved caspase 3 (#9661, Cell Signaling Technology), tubulin (#2148, Cell Signaling Technology) and GAPDH (#14C10, Cell Signaling Technology) at a concentration of 1:1000. This was followed by 1 h incubation with horse radish peroxidase-coupled secondary anti-rabbit IgG (#NA934V, GE Healthcare, München, Germany) or anti-mouse IgG (#SC-2005, Santa Cruz Biotechnologies, Heidelberg, Germany) at a concentration of 1:2000. Proteins were detected using ChemiDoc Touch Imaging System (Bio-Rad Laboratories, Hercules, CA, USA). Analyses were performed using ImageJ and Biorad Image lab software.

### TUNEL assay, immunostaining and ROS detection

INS-1E cells were seeded onto glass cover slips and cultured in the presence of test substances as indicated in each experiment. After incubation cells were fixed with 4% formalin.

For TUNEL (TdT-mediated dUTP-biotin nick end labeling) staining, fixed cells were permeabilized with 0.1% Triton X-100 in PBS and fractionated DNA was detected using the *In Situ* Cell Death Detection Kit (Roche Diagnostics, Basel, Switzerland). Nuclei were stained with 0.1 μg/ml 4’6-diamidino-2-phenylindole (DAPI). The percentage of TUNEL-positive cells was evaluated by counting at least 300 cells/condition/experiment.

For TXNIP protein detection, the cells were permabilised with 0.2% Triton X-100, blocked with 10% FCS-PBS and incubated overnight with anti-TXNIP antibody (1:100; rabbit; Abcam) followed by incubation with anti-rabbit IgG-Alexa Fluor488 for 1h. The nuclei were stained with 1 μM TOPRO3.

ROS production was measured using CellROX® Green Reagent (Thermo Fisher Scientific). Menadione (100 μM, Sigma) was used as positive control. CellROX Green Reagent (5 μM, Invitrogen) was added during a second hour of incubation. An increase in CellROX® signal in the nuclei and cytosol indicates an increase in oxidative stress.

Fluorescence was detected using a confocal microscope (Leica).

### TXNIP overexpression in INS-1E cells

INS-1E cells (2x10^4^/96-well) were cultured in RPMI 1640 after which, they were transfected with adenoviral TXNIP cDNA (Vector Biolabs, Malvern, PA, USA) at a MOI (multiplicity of infection) of 20. INS-1E cells transfected with an empty construct at the same MOI were used as controls. The transfection was performed for 24 h, after which the virus was removed. A thousand fold increase in basal TXNIP mRNA levels was achieved and the protein expression verified on western blot (S1 Fig). Acute insulin secretion was performed with the indicated test substances, 24 h post viral removal.

### Statistical analysis

Data are presented as mean ± SEM derived for the indicated number of independent experiments given in the figure legends. Statistical analysis was performed using Student’s unpaired t-test, when comparing between two treatment groups or ANOVA followed by Tukey’s post-test corrections, when comparing more than two treatment groups. Pearson’s correlation was used to evaluate the relationship between two variables and a linear regression curve for normally distributed data was plotted with the help of GraphPad Prism (Graphpad Software, Inc, La Jolla, CA, USA). Results with p<0.05 were considered statistically significant.

## Results

### Long chain fatty acids downregulate TXNIP mRNA levels independently of FFAR1/GPR40

**Fig 1:**
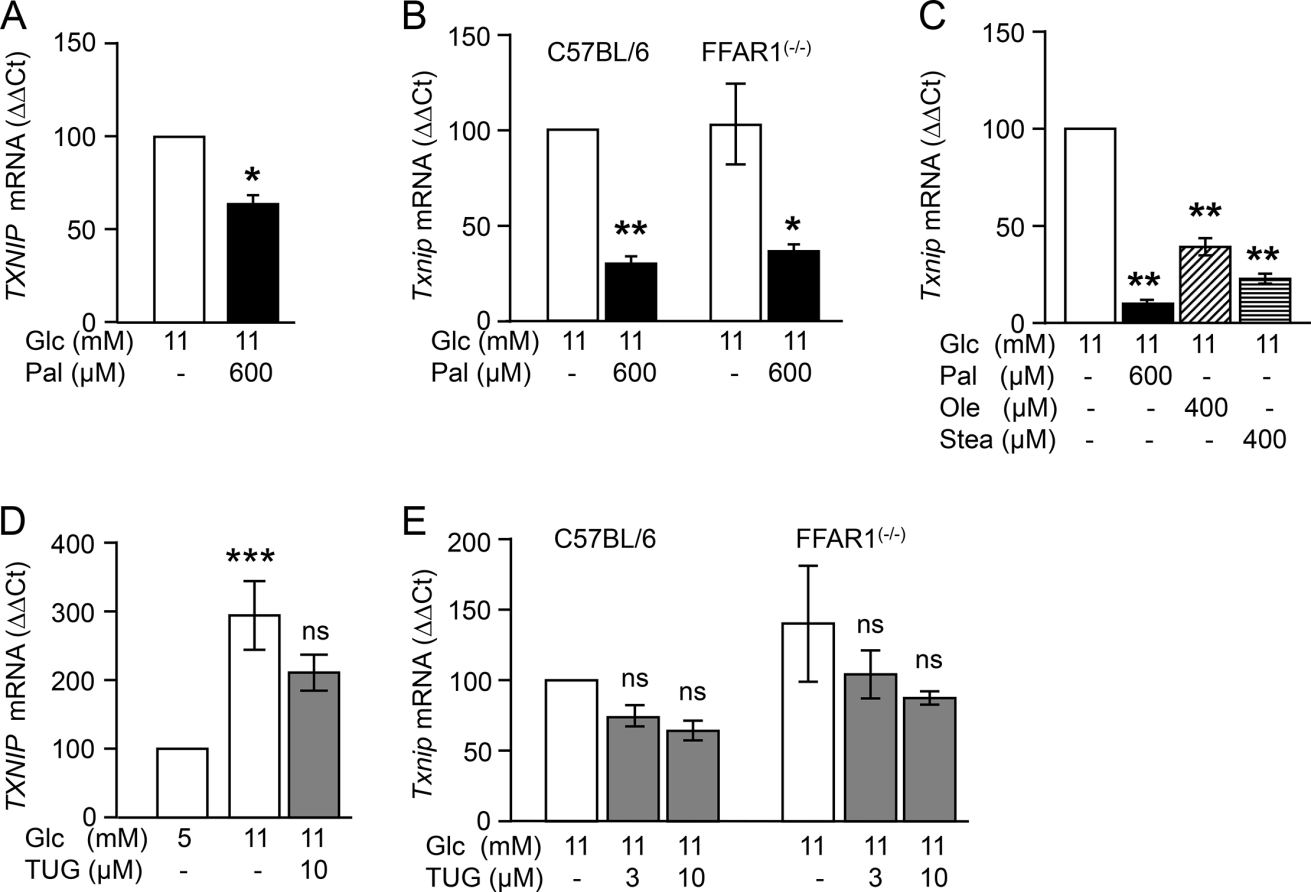
FFAR1/GPR40-independent effect of palmitate on TXNIP mRNA levels in human and mouse islets and INS-1E cells. (A and D) Isolated human islets, (B and E) mouse islets and (C) INS-1E cells were cultured for 24 h in the presence of test substances as indicated and described under Materials and methods. Changes in TXNIP mRNA levels (ΔΔCt) vs respective control (set to 100%, first column of each graph) are presented as mean ± SEM of n = 3–4 independent experiments; *p<0.05, **p<0.01, ***p<0.001 significant vs respective control. Abbreviations: Glc, glucose; Pal, palmitate; Ole, oleate; Stea, stearate; TUG, TUG-469 (FFAR1 agonist).

First, we examined whether the inhibitory effect of palmitate on TXNIP mRNA levels applies to human islets (Fig 1A). Exposure of isolated human islets to a lipotoxic concentration of palmitate (600 μM) for 24 h in the presence of 11 mM glucose significantly reduced the cellular concentration of TXNIP mRNA. As FFAR1/GPR40 contributes to the stimulatory effect of palmitate on insulin secretion, the role of FFAR1/GPR40 in the regulation of TXNIP mRNA was examined. Therefore, isolated human and *Ffar1*^(-/-)^ mice islets were treated with the FFAR1 agonist TUG-469 (Fig 1B, 1D and 1E) [18]. In human islets, TUG-469 did not significantly change TXNIP mRNA levels, whereas raising glucose from 5 mM to 11 mM induced an almost three-fold increase in TXNIP mRNA (Fig 1D). Likewise, in isolated mouse islets, palmitate significantly lowered TXNIP mRNA levels independent of the FFAR1/GPR40 expression, while the FFAR1/GPR40 agonist TUG-469 (3–10 μM) did not affect TXNIP mRNA (Fig 1B and 1E). The direct effect of palmitate on β-cell TXNIP mRNA is further validated in INS-1E cells. Other long chain fatty acids, i.e. oleate and stearate (both at 400 μM) mimicked the effect of palmitate (Fig 1C) and this is in agreement with a previous observation [17]. These results suggest that the palmitate-mediated downregulation of TXNIP mRNA occurs independently of FFAR1 activation.

### The effect of palmitate on TXNIP is glucose- and time-dependent

**Fig 2:**
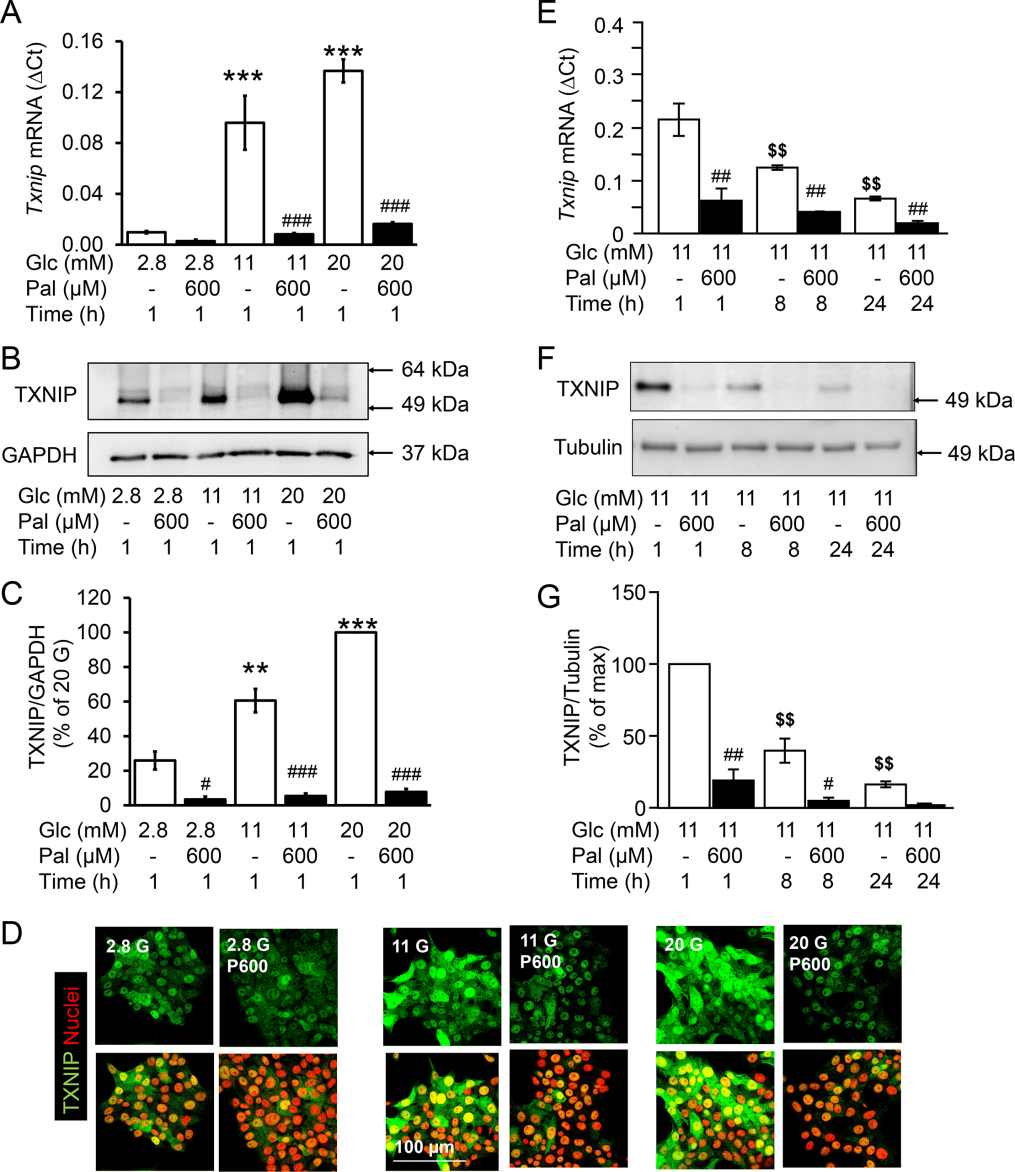
Palmitate- and time-dependent reduction of TXNIP expression despite stimulatory concentrations of glucose. INS-1E cells were cultured in the presence of test substances as indicated and described under Materials and methods. (A, E) Relative T*xnip* mRNA levels (ΔCt) to the house keeping gene (*Rps13*); (B, F) representative western blots; (C, G) quantitative analysis of western blots of three independent experiments presented as mean ± SEM; (D) Representative pictures of immunostained INS-1E cells for TXNIP (green) and nuclei (red). **p<0.01, ***p<0.001 vs 2.8 mM Glc, 1h; ^#^p<0.05, ^##^p<0.01, ^###^ p<0.001 significant effect of palmitate to the respective Glc concentration at the same time point; ^$ $^p<0.01 significant vs 11 mM Glc, 1h; Abbreviations: Glc, glucose; Pal, palmitate.

In order to understand the signalling pathways involved in TXNIP downregulation by palmitate in pancreatic β-cells, INS-1E cells were used in order to avoid the paracrine effects of other cell types within islets. INS-1E cells were exposed to lipotoxic concentrations of the saturated fatty acid palmitate (600 μM) under increasing glucose concentrations (2.8, 11 and 25 mM, Fig 2). Already after 1 h incubation, palmitate counteracted the glucose-induced upregulation of TXNIP mRNA and protein (Fig 2A–2D). Additionally, TXNIP mRNA declined over time and this time-dependent downregulation was significant in the presence of glucose, but not of palmitate (Fig 2E–2G). Since insulin and glucose exert opposing effects on TXNIP mRNA [20] and their concentrations may change during prolonged INS-1E cell culture, insulin and glucose concentrations in the culture medium were measured (S2 Fig). Insulin concentration in the medium increased over time and was further augmented by palmitate, reaching statistical significance at 8 h (S2 Fig). Glucose (11 mM) concentration was not altered after 8 h (10.7 mM) but was slightly lowered (9.7 mM) after 24 h. This minor decline of glucose in the culture medium as a result of cellular consumption cannot account for the pronounced time-dependent reduction of TXNIP mRNA levels.

These results demonstrate a dynamic regulation of TXNIP in β-cells: the glucose-induced increase is counteracted by palmitate and by at least one additional, time-dependent factor.

### The time-dependent downregulation of TXNIP is mediated by insulin and abrogated by the inhibition of histone deacetylase 1/2/3 (HDAC1/2/3)

**Fig 3:**
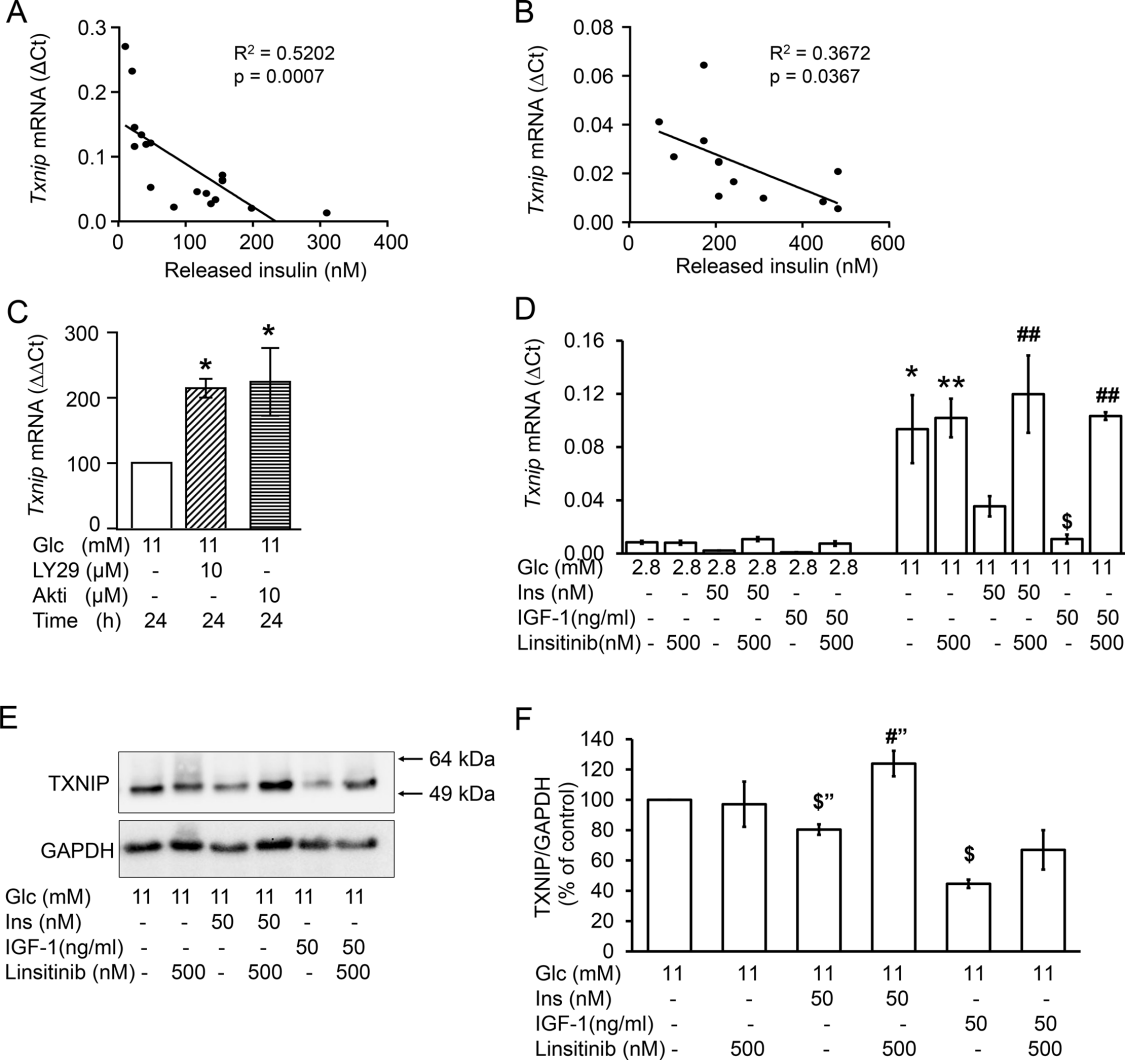
The time-dependent reduction of TXNIP mRNA levels correlates with insulin/IGF-1 receptor activation. (A, C-F) INS-1E cells and (B) isolated mouse islets were cultured in the presence of test substances as indicated and described under Materials and methods. (A and B) Correlation of insulin released into the medium with TXNIP mRNA levels of (A) INS-1E cells and (B) mouse islets; (C) Changes of TXNIP mRNA levels (ΔΔCt) vs control (11 mM glucose at 24 h) set to 100%; (D-F) The IR/IGF1R inhibitor linsitinib was applied for 2 h, insulin/IGF-1 the second hour of incubation; (D) Relative *Txnip* mRNA levels (ΔCt) to the house keeping gene (*Rps13*). (E) Representative western blot of TXNIP and (F) quantitative analysis of three independent experiments; GAPDH was used as loading control. Results are presented as mean ± SEM of n = 3–4 independent experiments. *p<0.05, **p<0.01 significant vs 2.8 mM Glc; $p<0.05 significant vs 11 mM Glc; ^**#**^p<0.05, ^**##**^p<0.01 significant effect of linsitinib (“, unpaired t-test). Abbreviations: Glc, glucose; Ins, insulin; LY29, LY294002 (PI3K inhibitor); Akti, AKT/PKB inhibitor.

Previous observations suggest that insulin counteracts glucose-induced upregulation of TXNIP [20]. In agreement, the concentration of insulin which accumulated in the medium displayed a negative association with cellular levels of TXNIP mRNA in INS-1E cells and mouse islets (Fig 3A and 3B). The inhibition of PI3K by LY294002 (10 μM) and that of AKT/PKB by Akti (10 μM) significantly augmented TXNIP mRNA levels at 24 h (Fig 3C). The concomitant increase of AKT/PKB phosphorylation confirmed that insulin activated IR/IGF-1R signalling pathway (S3 Fig). Acute addition of insulin or IGF-1 to the medium reduced TXNIP mRNA and protein levels (Fig 3D–3F). The insulin and IGF-1 receptor antagonist linsitinib counteracted the effect of insulin and IGF-1 on TXNIP mRNA and protein (Fig 3D–3F). IGF-1 was tested since high concentrations of insulin crossreact and activate IGF-1R (25).

**Fig 4:**
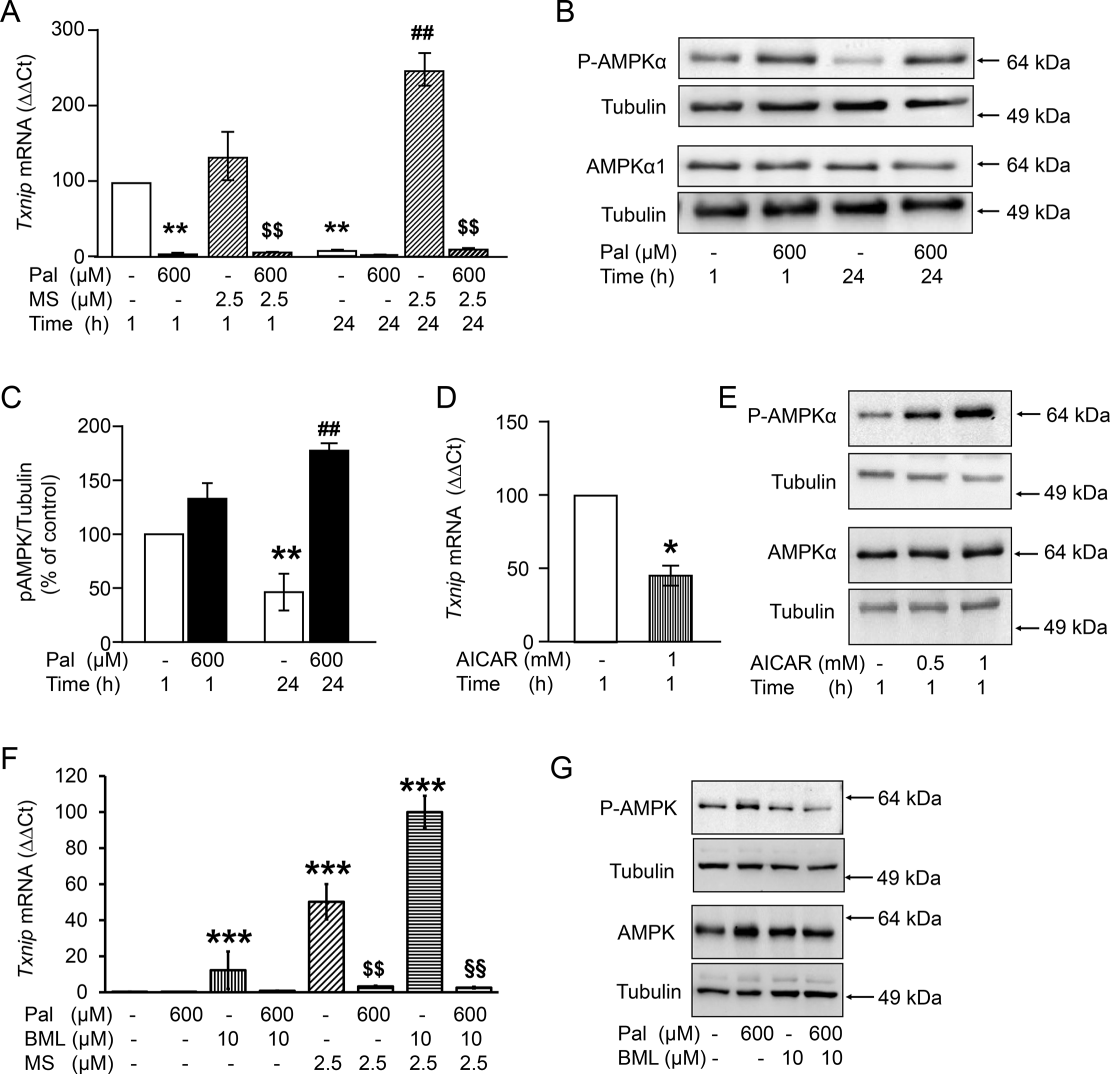
Inhibition of histone deacetylases antagonizes the time-dependent decline of TXNIP without reversing the effect of palmitate. INS-1E cells were cultured in the presence of 11 mM glucose and test substances as indicated and described under Materials and methods. (A,D,F) Changes of TXNIP mRNA levels (ΔΔCt) vs control set to 100% in A and D; vs max effect in F. (B,E,G) Representative western blots of ^172^Thr-P-AMPKα and AMPKα1; Tubulin is used as loading control. (C) Quantitative analysis of western blots of three independent experiments of B. Results are given as mean ± SEM of n = 3–4 independent experiments. *p<0.05, **p<0.01, *** p<0.001 denotes significance to control, i.e. 11 mM Glc at 1h; ^**##**^p<0.01 to 11 mM Glc at 24 h. $ $p<0.01 significant effect of palmitate in the presence of MS. §§p<0.01 significant effect of palmitate in the presence of MS and BML. Abbreviations: Glc, glucose; Pal, palmitate; MS, MS-275 (HDAC1/2/3 inhibitor); BML, BML-275 (AMPK inhibitor); AICAR, AMPK activator.

Next, we tested the hypothesis that the inhibitory effect on TXNIP mRNA was mediated by HDAC, a downstream target of PI3K/AKT pathway [21]. The time-dependent downregulation of TXNIP was completely abrogated when INS1-E cells were cultured in the presence of the HDAC1/2/3 inhibitor MS-275 (2.5 μM, Fig 4A). In contrast, the inhibitory effect of palmitate remained unchanged suggesting that two independent mechanisms transmit the insulin- and palmitate-dependent inhibition of TXNIP expression.

### Effects of AMPK on TXNIP mRNA levels and its regulation by palmitate

Evidence has been presented that palmitate-induced inhibition of TXNIP expression is at least partially mediated via the stimulation of AMPK [17]. In agreement, palmitate increased, whereas glucose decreased ^172^Thr-phosphorylation of AMPKα at 24 h (Fig 4B and 4C). AICAR, the synthetic agonist of AMPK, mimicked the effect of palmitate and decreased TXNIP mRNA levels by 50% while increasing concentration dependently ^172^Thr-phosphorylation of AMPKα (Fig 4D and 4E). To assess whether AMPK is involved in palmitate-mediated downregulation of TXNIP mRNA, AMPK activity was inhibited with BML-275 (10 μM). BML-275 was unable to reverse the effect of palmitate on TXNIP, despite inhibiting palmitate-induced AMPK phosphorylation (Fig 4F and 4G). In the presence of BML-275 and MS-275, i.e. the inhibition of AMPK and HDAC1/2/3, TXNIP mRNA achieved the highest level, but was still downregulated by palmitate (Fig 4F). This results suggest that neither HDAC1/2/3 nor AMPK mediate the effect of palmitate on TXNIP mRNA.

### Palmitate induces β-cell death despite downregulation of TXNIP

**Fig 5:**
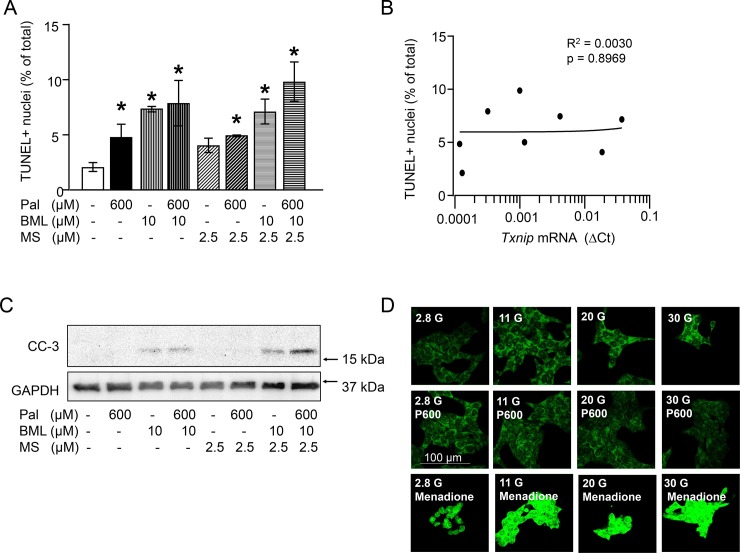
Changes in TXNIP mRNA levels correspond to ROS but not to changes in INS-1E cell death. (A-C) INS-1E cells were cultured for 24 h in the presence of 11 mM glucose and test substances as indicated and described under Materials and methods. (A) Percentage of TUNEL positive cells, *p<0.05 significant vs control culture, (B) correlation between TUNEL positive nuclei and relative TXNIP mRNA levels (∆Ct), (C) representative western blot for cleaved caspase 3, GAPDH was used as loading control; (D) representative pictures of INS-1E cells stained with the ROS sensor CellROXGreen. INS-1E cells were cultured for 2 h in the presence of different glucose concentrations, palmitate (600 μM) and menadione (100 μM; ROS inducer) as indicated. CellROXGreen (5 μM) was applied for 1 h in culture. Note the increased green fluorescence (increased ROS levels) in the cells exposed to 30 mM Glc or menadione vs 2.8 mM glucose and 30 mM Glc+palmitate. Abbreviations: Pal, P600, palmitate; BML, BML-275 (AMPK inhibitor); CC-3, cleaved caspase-3.

Apoptotic β-cell death and ROS production were quantified in order to understand whether TXNIP modulation under glucolipotoxic conditions may have a functional consequence (Fig 5). As expected, palmitate increased apoptosis, however, the highest rate of apoptosis (TUNEL positive nuclei and cleaved caspase 3) was detected upon inhibition of AMPK and HDAC1/2/3 under palmitate exposure, a condition where TXNIP mRNA level was the lowest (Fig 5A and 5C and Fig 4F). Consequently, the apoptotic rate did not correlate to changes in TXNIP mRNA levels (Fig 5B). Furthermore, the inhibition of palmitate-stimulated kinases, JNK by SP600125, ERK1/2 by PD98059 and PKCα/β by Gö6976 did not antagonize the effect of palmitate on TXNIP mRNA levels (S3 Fig). Inhibition of JNK, however, reversed palmitate-induced phosphorylation of cJun and apoptotic cell death (S3 Fig). On the contrary, palmitate diminished glucose-induced ROS production as suggested by the reduced green fluorescence of the ROS sensor CellROX Green in the presence of 20 mM or 30 mM glucose and palmitate (Fig 5D). This results suggest that TXNIP downregulation is irrelevant for the palmitate induced cell death but it contributes to attenuation of glucose-induced oxidative stress by palmitate.

### Palmitate-mediated TXNIP downregulation does not influence insulin secretion

**Fig 6:**
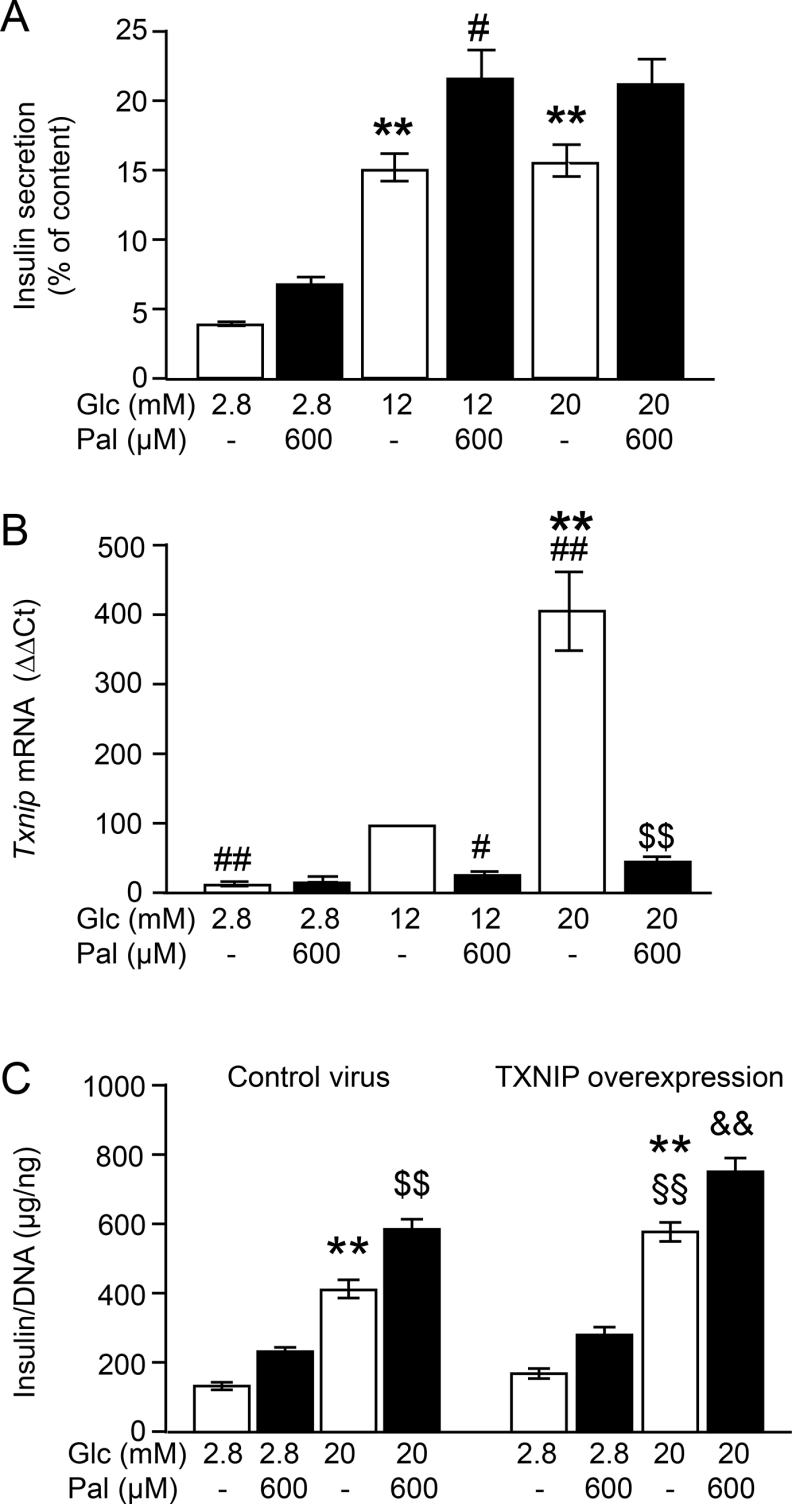
Palmitate-mediated augmentation of GSIS occurs independently of TXNIP mRNA regulation. INS-1E cells were incubated for 1 h in KRB supplemented with test substances as described under Materials and methods. (A and B) Parallel measurement of (A) insulin secretion and (B) changes of TXNIP mRNA levels (ΔΔCt). (C) Insulin secretion of INS-1E cells after transient transfection with TXNIP or control virus. Data are expressed as mean ± SEM of n = 3–4 independent experiments. **p<0.01 denotes significance to the respective 2.8 mM Glc; ^**#**^p<0.05, ^**##**^p<0.01 to 12 mM Glc; ^**$ $**^p<0.01 to 20 mM Glc; ^**§§**^p<0.001 to control virus transfected cells at 20 mM Glc; ^**&&**^p<0.01 significant effect of palmitate vs 20 mM Glc in TXNIP overexpressing cells. Abbreviations: Glc, Glucose, Pal, palmitate.

Finally, in order to understand whether the regulation of TXNIP mRNA levels by palmitate translated to changes in insulin secretion, we assessed TXNIP mRNA levels in parallel to acute stimulation of insulin secretion by glucose and palmitate in KRB (Fig 6A and 6B). Insulin secretion was stimulated 3–4 fold by increasing glucose from 2.8 to 12 mM (Fig 6A). This stimulation was accompanied by a 5-fold increase in TXNIP mRNA levels (Fig 6B). At 20 mM glucose insulin secretion was not higher than that at 12 mM glucose but TXNIP mRNA levels displayed a further 4-fold increase. Palmitate augmented insulin secretion at 12 and 20 mM glucose while reducing TXNIP mRNA levels by 80–90% (Fig 6A and 6B). In order to resolve the opposing regulatory patterns of TXNIP mRNA and insulin secretion, TXNIP was transiently overexpressed in INS-1E cells, as described in detail in the Materials and methods (S1 Fig). Glucose-induced insulin secretion was significantly higher in TXNIP-overexpressing cells but palmitate-mediated augmentation of GSIS, however, remained unchanged (Fig 6C).

These results suggest that palmitate-mediated downregulation of TXNIP does not contribute to the augmentation of GSIS.

**Fig 7:**
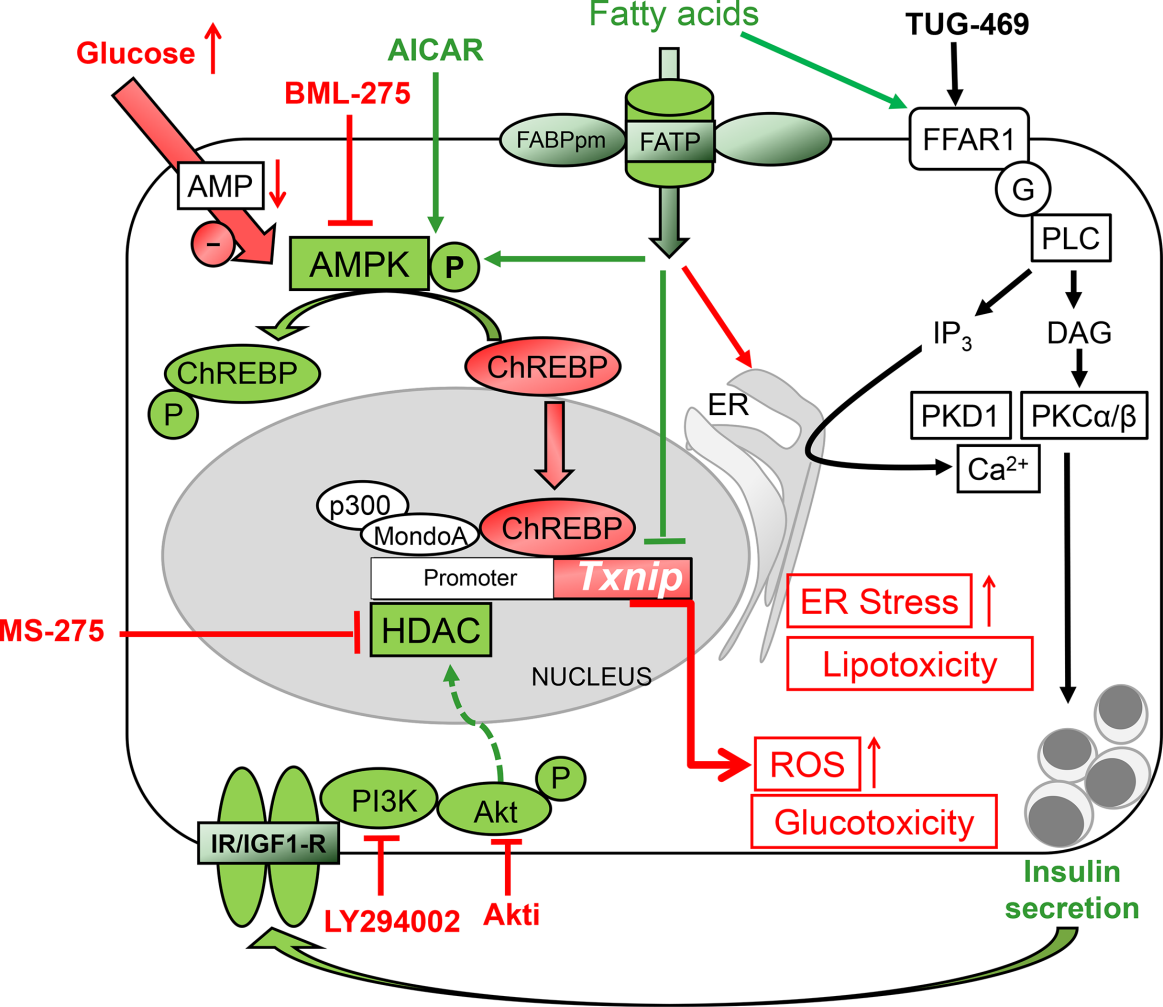
Regulation of TXNIP mRNA levels in β-cells. Transcription of TXNIP is under the control of a protein complex consisting of ChREBP (carbohydrate-responsive element-binding protein) and MondoA as well as the histone acetyltransferase P300. ChREBP is negatively regulated by AMPK. Consequently, stimulation of AMPK by AICAR inhibits ChREBP and TXNIP expression, while inhibition of AMPK by glucose and BML-275 activates ChREBP and increases TXNIP mRNA levels. Histone deacetylase 1 (HDAC1), counteracts P300-mediated histone acetylation, and is involved in insulin-mediated downregulation of TXNIP expression. Thus, inhibition of PI3K, AKT or HDAC1/3 increases TXNIP mRNA levels. Fatty acids-mediated stimulation of insulin secretion occurs via FFAR1/GPR40, a signalling pathway not involved in regulation of TXNIP expression. Fatty acids activate AMPK and have an additional effect on TXNIP mRNA levels. Fatty acids counteract glucose-induced TXNIP expression and ROS elevation, events which do not impede the ER strees-associated lipotoxic effect.

To summarize, our data show that glucose-mediated upregulation of TXNIP is efficiently antagonized by palmitate and insulin. The palmitate effect is independent of FFAR1, i.e. the signalling pathway which augments GSIS, while the insulin effect involves activation of HDAC1/2/3 (Fig 7).

## Discussion

This study provides evidence that fatty acids stabilize the oxidative defense of β-cells via inhibition of TXNIP expression in isolated human islets, mouse islets and INS-1E cells. The effect of palmitate was not mediated through FFAR1/GPR40 although saturated (palmitate and stearate) and unsaturated (oleate) long chain fatty acids, both endogenous agonists of FFAR1/GPR40, reduced TXNIP mRNA levels. Palmitate lowered TXNIP mRNA in FFAR1 deficient islets and the selective FFAR1 agonist TUG-469 did not mimic the effect of palmitate. In contrast, a previous study suggested an inhibitory effect of the FFAR1 agonist CNX-011-67 on TXNIP mRNA levels in the presence of 16.7 mM glucose and 500 μM palmitate [22]. This observation could be explained by an off-target effect of the agonist, since FFAR1 was already fully activated by 500 μM palmitate. In a study by Shaked *et al*., it was concluded that the effect of palmitate on TXNIP mRNA was mediated by AMPK [17]. Indeed, palmitate stimulates AMPK phosphorylation, and AICAR, an AMPK stimulator, mimicked the effect of palmitate on TXNIP mRNA. The AMPK inhibitor BML-275 upregulated TXNIP mRNA, alone as well as in combination with the HDAC1/3 inhibitor MS-275 (Fig 4F). However, the palmitate effect on TXNIP persisted despite inhibition of the enzymes involved in transcriptional regulation (AMPK and HDAC1/3), suggesting a distal, possibly post-transcriptional modulation of TXNIP mRNA levels, i.e. TXNIP mRNA degradation. When mRNA synthesis was inhibited by actinomycin D (5 μg/ml) TXNIP mRNA was largely reduced. Under this condition palmitate did not affect the rate of TXNIP mRNA degradation (data not shown). These observations lead to the conclusion that palmitate interferes with TXNIP mRNA synthesis rather than degradation.

In addition to palmitate, insulin also counteracts glucose-mediated upregulation of TXNIP. Exogenously added insulin reduced, while inhibitors of insulin/IGF-1 receptor signalling, i.e. LY294002 and Akti, augmented TXNIP mRNA levels. Moreover, the IR/IGF-1R antagonist linsitinib revoked the inhibitory effects of insulin and IGF-1 on TXNIP mRNA and protein (Fig 3D–3G). These observations suggested an autocrine action of insulin via the activation of β-cell insulin/IGF-1 receptors [23–25]. The effect of insulin was completely abrogated by the HDAC inhibitor MS-275. This selective abrogation of the time-dependent decline clearly dissociates the insulin-mediated effect from the palmitate effect and provides strong evidence that two independent signalling pathways counteract glucose stimulation of TXNIP expression.

A functional consequence of this regulation is likely since the fast (within 1 h) and long-lasting (24 h) changes in TXNIP mRNA levels directly translate to changes in TXNIP protein. However, alterations in TXNIP mRNA levels showed no correlation with palmitate-induced apoptosis although palmitate reduced ROS production. In agreement with this, a previous study has shown that *Txnip*^(-/-)^ mice are not protected from palmitate-mediated apoptosis [26]. Thus, we conclude that TXNIP is not actively involved in regulating or modulating palmitate-mediated β-cell death [19].

In order to understand whether palmitate-induced TXNIP downregulation contributes to its effect on secretion, the endogenous regulation of TXNIP was overridden by a transient overexpression of TXNIP. Although GSIS was significantly higher in TXNIP-overexpressing cells the stimulatory effect of palmitate was not altered. In conclusion, our data rule out a major role for TXNIP in palmitate-dependent insulin secretion and lipotoxicity.

A role for TXNIP in mitochondrial fuel metabolism has been suggested in heart and skeletal muscle, both of which utilize fatty acids, i.e. β-oxidation, as energy source. In the heart, TXNIP deficiency was linked to an increase in β-oxidation promoting enzymes [27]. On the other hand, in the skeletal muscle, TXNIP deficiency led to a downregulation of enzymes involved in β-oxidation [28]. Interestingly, in the skeletal muscle TXNIP expression is not downregulated upon exposure to fatty acids [29]. In contrast to these two tissues, pancreatic islets do not depend on fatty acids as an energy source and therefore, β-oxidation may play a minor role in metabolism. In agreement, palmitate did not augment mitochondrial respiration in INS-1E cells [30]. Therefore, we would like to speculate that one possible consequence of TXNIP downregulation in β-cells under exposure to fatty acids might be an interference with the mitochondrial fuel handling and metabolism. This concept needs further evaluation.

In summary, we present experimental evidence that long chain fatty acids and insulin efficiently counteract glucose-induced TXNIP expression via distinct mechanisms. Thus, under hyperglycemia, fatty acids and insulin reduce oxidative stress in β-cells.

## Supporting information

S1 Fig Palmitate-induced reduction of TXNIP mRNA levels is independent of PKCα/β, ERK and JNK signalling. (PDF)

S2 Fig Concentration of glucose and accumulation of secreted insulin during INS-1E cell culture. (PDF)

S3 Fig Palmitate-induced reduction of TXNIP mRNA levels is independent of PKCα/β, ERK and JNK signalling. (PDF)

S1 Table List of primers used for qRT-PCR. (PDF)

## Abbreviations

AICAR: 5-Aminoimidazole-4-carboxamide ribonucleotide
AMPK: 5' adenosine monophosphate-activated protein kinase
BSA: bovine serum albumin
ChREBP: carbohydrate-responsive element-binding protein
ERK1/2: extracellular signal regulated kinase 1/2
GPR40/FFAR1: G-protein coupled receptor 40/fatty acid receptor 1
GSIS: glucose stimulated insulin secretion
HDAC: histone deacetylase
IR: insulin receptor
IGF-1R: insulin growth factor 1 receptor
JNK: c-Jun N-terminal kinase
KRB: Krebs Ringer buffer
MOI: multiplicity of infection
NEFAs: non-esterified fatty acids
PI3K: phosphatidylinositol-4,5-bisphosphate 3-kinase
PKA: protein kinase A
PKB/Akt: protein kinase B
PKC: protein kinase C
TUNEL: TdT-mediated dUTP-biotin nick end labeling
TXNIP: thioredoxin interacting protein
WT: wild type
