# Supplementary material for: Palmitate and insulin counteract glucose-induced thioredoxin interacting protein (TXNIP) expression in insulin secreting cells via distinct mechanisms
Source: PLoS One. 2018 May 29;13(5):e0198016. doi: 10.1371/journal.pone.0198016 (PMC5973613; doi:10.1371/journal.pone.0198016)
Supplement: S1 Fig — (PDF) [file pone.0198016.s001.pdf]

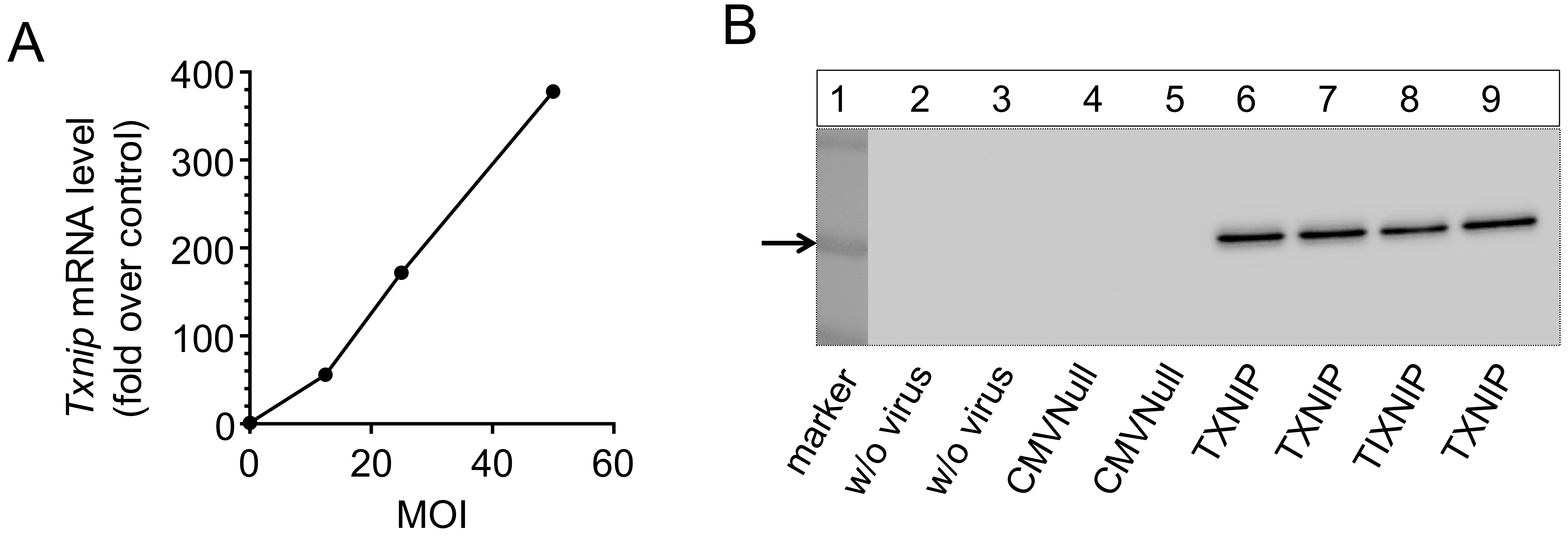

S1 Fig. Overexpression of *TXNIP* in INS-1 E cells. INS-1E cells were cultured and transfected as described under Material and methods. (A) Relative *TXNIP* mRNA (fold change over control, untransfected cells) and (B) western blot for TXNIP showing TXNIP overexpression in transfected cells. Marker at 50 kDa.
