# Supplementary material for: Palmitate and insulin counteract glucose-induced thioredoxin interacting protein (TXNIP) expression in insulin secreting cells via distinct mechanisms
Source: PLoS One. 2018 May 29;13(5):e0198016. doi: 10.1371/journal.pone.0198016 (PMC5973613; doi:10.1371/journal.pone.0198016)
Supplement: S2 Fig — (PDF) [file pone.0198016.s002.pdf]

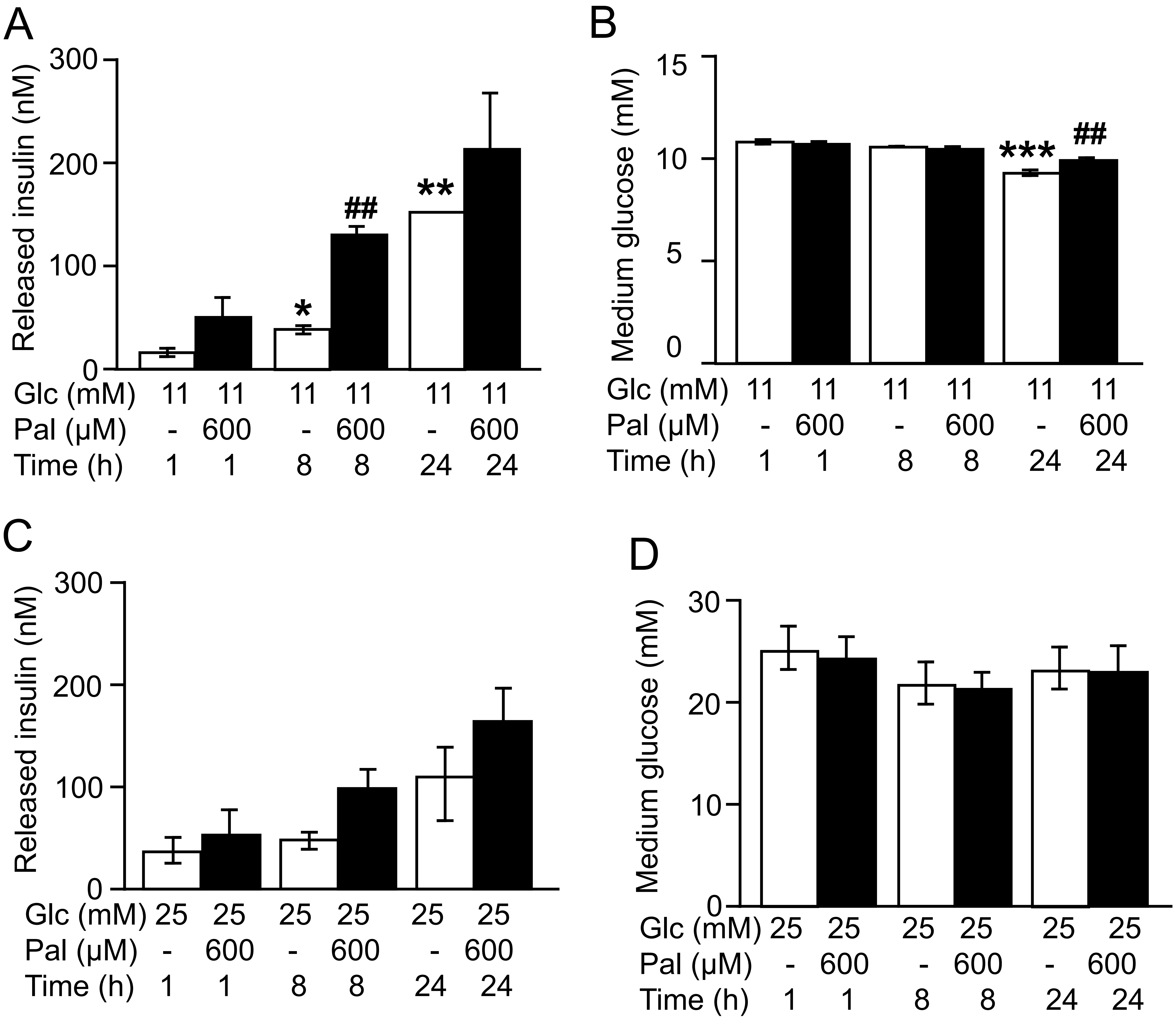

S2 Fig. Concentration of glucose and accumulation of secreted insulin during INS-1E cell culture. INS-1E cells were cultured in the presence of test substances as indicated and described under Materials and methods. (A, C) insulin and (B, D) glucose concentrations of the medium after culture time as indicated expressed as mean ± SEM of n=4-6 independent experiments. *p<0.05, **p<0.01, ***p<0.001 vs 11 mM Glc 1h; ##p<0.01 significant effect of palmitate to the respective Glc concentration at the same time point; Abbreviations: Glc, glucose; Pal, palmitate.
