# Supplementary material for: Palmitate and insulin counteract glucose-induced thioredoxin interacting protein (TXNIP) expression in insulin secreting cells via distinct mechanisms
Source: PLoS One. 2018 May 29;13(5):e0198016. doi: 10.1371/journal.pone.0198016 (PMC5973613; doi:10.1371/journal.pone.0198016)
Supplement: S3 Fig — (PDF) [file pone.0198016.s003.pdf]

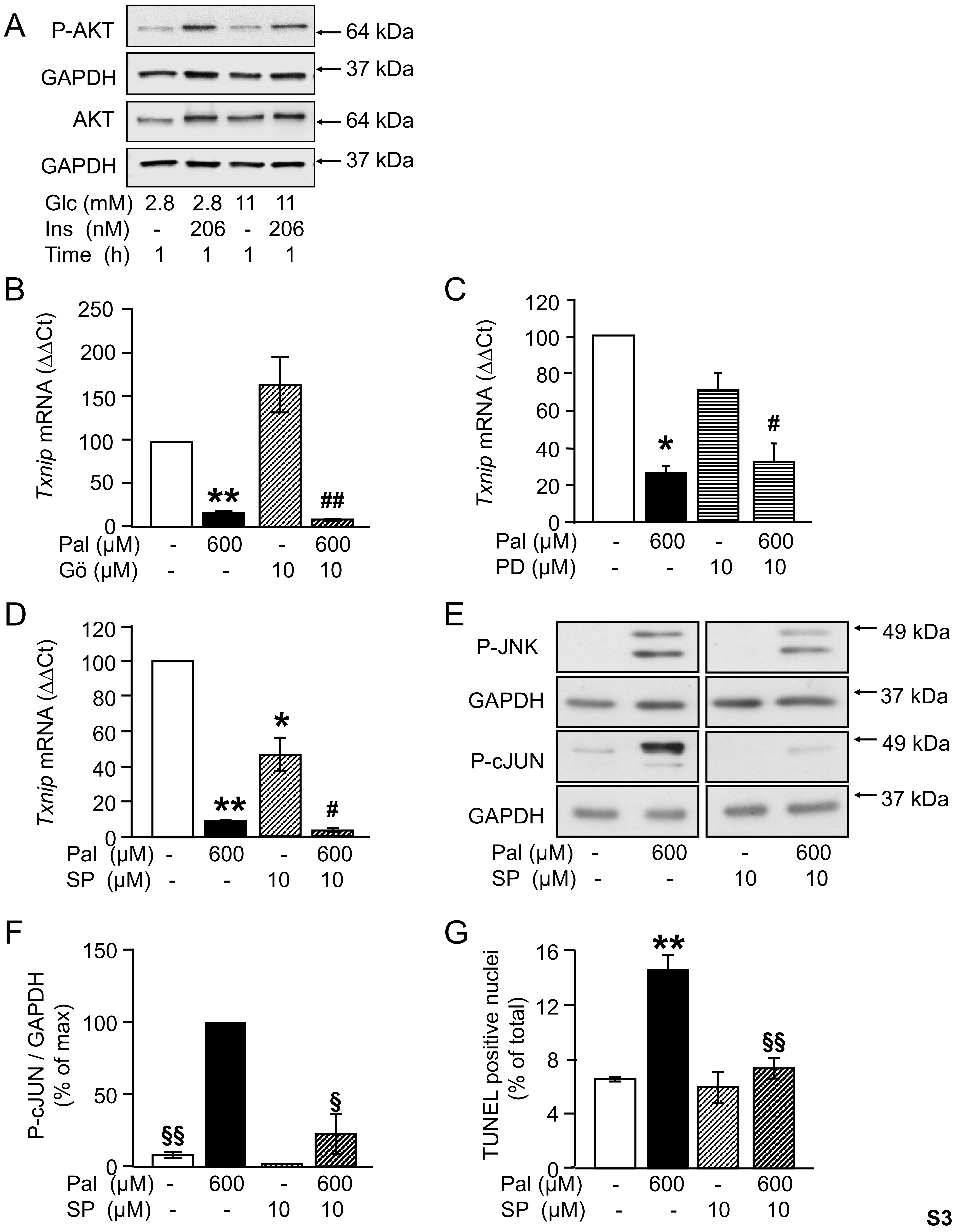

A
P-AKT
GAPDH
AKT
GAPDH
64 kDa
37 kDa
64 kDa
37 kDa
Glc (mM) 2.8 2.8 11 11
Ins (nM) - 206 - 206
Time (h) 1 1 1 1
B
Txnip mRNA (ΔΔCt)
Pal (μM) - 600 - 600
Gö (μM) - - 10 10
C
Txnip mRNA (ΔΔCt)
Pal (μM) - 600 - 600
PD (μM) - - 10 10
D
Txnip mRNA (ΔΔCt)
Pal (μM) - 600 - 600
SP (μM) - - 10 10
E
P-JNK
GAPDH
P-cJUN
GAPDH
49 kDa
37 kDa
49 kDa
37 kDa
Pal (μM) - 600 - 600
SP (μM) - - 10 10
F
P-cJUN / GAPDH (% of max)
Pal (μM) - 600 - 600
SP (μM) - - 10 10
G
TUNEL positive nuclei (% of total)
Pal (μM) - 600 - 600
SP (μM) - - 10 10

S3 Fig

S3 Fig. Palmitate-induced reduction of TXNIP mRNA levels is independent of PKCα/β, ERK and JNK signalling. INS-1E cells were cultured in the presence of (A) 2.8 mM or (B-G) 11 mM glucose and test substances for (A) 1 h or (B-G) 24 h. (A) Representative western blot of ^{473}Ser-P-AKT and AKT. (B-D) Changes of TXNIP mRNA levels (ΔΔCt) vs control (first column in each graph) set to 100 %. (E, F) Representative western blots and quantitative analysis of n=3 independent experiments of ^{183}Thr/^{185}Tyr-P-JNK and ^{73}Ser-P-cJUN. GAPDH was used as the loading control. (G) Cell death was evaluated by the percentage of TUNEL-positive cells. Results are expressed as mean ± SEM of n=3-4 independent experiments. *$p<0.05$, **$p<0.01$ denotes significance to control (first bar of each graph); $^{\#}p<0.05$, $^{\#\#}p<0.01$ indicates a significant Pal effect in the presence of inhibitor (Gö, PD or SP); $^{\S}p<0.05$, $^{\S\S}p<0.01$ indicates significance to Pal. Abbreviations: Pal, palmitate; Gö, Gö6976 (PKCα/β inhibitor); PD, PD98059 (ERK1/2 inhibitor); SP, SP600125 (JNK inhibitor).
