# Supplementary material for: Palmitate and insulin counteract glucose-induced thioredoxin interacting protein (TXNIP) expression in insulin secreting cells via distinct mechanisms
Source: PLoS One. 2018 May 29;13(5):e0198016. doi: 10.1371/journal.pone.0198016 (PMC5973613; doi:10.1371/journal.pone.0198016)
Supplement: S1 Table — (PDF) [file pone.0198016.s004.pdf]

S1 Table: Primers and probes used for qRT-PCR

Species	Gene	Roche probe	Upstream primer	Downstream primer
**Human**	*RPS13*	#68	5’-CCCCACTTGGTTGAAGTTGA-3’	5’-ACACCATGTGAATCTCTCAGGA-3’
	TXNIP	#3	5‘-GGCTAAAGTGCTTTGGATGC-3‘	5‘-TGATCACCATCTCATTCTCACC-3‘
**Mouse**	*Rps13*	#110	5‘-TGCTCCCACCTAATTGGAAA-3‘	5‘-CTTGTGCACACAACAGCATTT-3‘
	Txnip	*#62*	5’-CAAAGTCGAATACTCCTTGCTGA-3’	5’-CTGAATGTCCGGCTGCTC-3’
**Rat**	*Rps13*	#12	5‘-CTGACGACGTGAAGGAACAA-3‘	5‘-TCACAAAACGGACCTGTGC-3‘
	Txnip	*#20*	5’-GGGTGAAGGCTTTTCTCGAT-3’	5’-CCTTTTTGGCAGACACTGGT-3’
